# Fecal Microbiota Transplantation in Dogs and Cats: Evidence for Gastrointestinal and Emerging Extra-Intestinal Applications

**DOI:** 10.3390/ani16172744

**Published:** 2026-09-02

**Authors:** Jian Jin, Chao Xu, Wenbin Bao

**Affiliations:** 1 College of Animal Science and Technology, Yangzhou University, Yangzhou 225009, China; 009027@yzu.edu.cn (J.J.); mx120200807@stu.yzu.edu.cn (C.X.); 2 Institute of Comparative Medicine, College of Veterinary Medicine, Yangzhou University, Yangzhou 225009, China

**Keywords:** fecal microbiota transplantation, dog, cat, companion animals, chronic enteropathy, dysbiosis, microbiome, donor screening

## Abstract

Fecal microbiota transplantation (FMT) is an emerging microbiome-directed intervention for dogs and cats, but its clinical role remains incompletely defined. This structured narrative review integrates peer-reviewed clinical, mechanistic, methodological, and safety evidence on companion-animal FMT published between 2005 and 2026, with emphasis on efficacy, tolerability, mechanisms, product preparation, donor screening, and research priorities. In dogs, the most consistent clinical signal concerns chronic enteropathy (CE), where observational studies frequently report reduced disease activity scores after FMT, whereas small randomized trials have shown mixed results; however, causal inference remains limited by small sample sizes, heterogeneous diagnostic criteria and treatment protocols, and concurrent therapies. The strongest controlled canine signal comes from parvoviral enteritis, where adjunctive enema-based FMT accelerated diarrhea resolution and shortened hospitalization. Feline data support short-term tolerability and measurable microbiome activity, but clinical efficacy remains preliminary; notably, the first controlled feline chronic enteropathy (CE) trial showed no significant improvement in dysbiosis index or clinical activity scores compared with controls. Proposed mechanisms include donor microbial engraftment, metabolic restoration, immune modulation, and gut–brain and gut–skin axis signaling, although veterinary-specific validation remains limited. Standardized donor screening, batch-level quality control, dose-finding studies, long-term safety surveillance, and adequately powered sham-controlled trials are the main prerequisites for responsible clinical translation.

## Simple Summary

Chronic digestive problems are common in dogs and cats and can be difficult to manage. Many affected animals have an imbalance in the community of microbes that normally live in their intestines. Fecal microbiota transplantation is a treatment in which stool from a carefully screened healthy donor is processed and given to a sick animal to help restore a healthier balance of gut microbes. This review summarizes current evidence on the use of this treatment in pet dogs and cats. The evidence is most developed in dogs with long-term bowel disease, where several studies report fewer signs such as diarrhea, although many studies were uncontrolled and the animals received other treatments at the same time. In cats, the available studies suggest the procedure is generally well tolerated, but clear proof of clinical benefit is still limited. The review also explains possible ways it works, including donor microbes settling in the gut, recovery of helpful substances made by gut bacteria, effects on the immune system, and signaling between the gut and other body systems. Before this treatment can be widely used in veterinary practice, donor screening, product preparation, dosing, and long-term safety still need to be better standardized.

## 1. Introduction

The gastrointestinal microbiome of companion animals is a metabolically and immunologically active ecosystem that contributes to nutrient processing, mucosal barrier integrity, colonization resistance, bile acid conversion, and immune development [1,2,3,4,5,6]. In dogs and cats, disruption of this ecosystem—commonly described as dysbiosis—is associated with chronic gastrointestinal enteropathies, antibiotic-associated diarrhea, and selected extra-intestinal disorders [2,7]. Dysbiosis is not only a compositional abnormality but also a functional disturbance characterized by altered short-chain fatty acid production, impaired bile acid metabolism, reduced colonization resistance, and expansion of facultative pathogens [2,8,9,10,11]. Chronic gastrointestinal disorders are common reasons for veterinary consultation and referral. In dogs, recent ACVIM-endorsed recommendations favor the term chronic inflammatory enteropathy (CIE) over inflammatory bowel disease (IBD), because canine CIE is not considered directly equivalent to human IBD [12]. However, chronic enteropathy (CE) is a broader clinical term encompassing dogs with persistent or recurrent gastrointestinal signs, including populations in which intestinal biopsy and histopathological confirmation of inflammation may not be available. Because the canine FMT literature reviewed here includes both histopathologically confirmed inflammatory enteropathy and broader chronic enteropathy populations, we use CE as the overarching term throughout the clinical evidence synthesis. CIE is reserved for histopathologically supported inflammatory enteropathy, for terminology specifically defined by current consensus guidance, or for study populations explicitly characterized as CIE. IBD is retained only where it reflects terminology used in the original publication or established disease activity indices.

Canine chronic enteropathy comprises heterogeneous and multifactorial disease phenotypes involving interactions among diet, the intestinal microbiota, mucosal immunity, environmental factors, and host susceptibility. Canine chronic enteropathies have traditionally been categorized according to treatment response as food-responsive enteropathy (FRE), antibiotic-responsive enteropathy (ARE), immunosuppressant-responsive enteropathy (IRE), and non-responsive enteropathy (NRE). However, recent advances have challenged this framework. In particular, a refined classification has proposed replacing ARE with microbiota-related modulation-responsive enteropathy (MrMRE), reflecting decreasing reliance on empirical antimicrobial therapy and increasing use of microbiota-directed approaches, including prebiotics, probiotics, postbiotics, dietary modulation, and fecal microbiota transplantation (FMT) [13]. More recent ACVIM-endorsed guidance continues to emphasize phenotyping according to treatment response, including food-responsive CIE (CIE-FR) and immunosuppressant-responsive CIE (CIE-IR) [12]. Although clinical signs may improve following dietary, microbiota-directed, or immunomodulatory interventions, underlying microbial and metabolic abnormalities may persist [2,8,14,15,16,17,18,19]. In cats, chronic enteropathy (CE) remains the broader clinical term, and the diagnostic overlap between inflammatory chronic enteropathies and small-cell intestinal lymphoma creates additional complexity [20,21]. FMT involves the transfer of processed fecal material from a healthy, screened donor to a recipient with disease. In human medicine, FMT is best established for recurrent *Clostridioides difficile* infection [22,23,24,25,26,27,28], with additional but more variable evidence in inflammatory bowel disease, including ulcerative colitis, and in metabolic disorders [29,30,31,32,33,34,35,36,37,38]. The application of FMT in veterinary medicine, however, requires caution because dogs and cats differ from humans in diet, gastrointestinal anatomy, bile acid profiles, core microbial taxa, pathogen risks, and regulatory context. Veterinary FMT should therefore be evaluated on the basis of species-specific clinical evidence rather than by direct extrapolation from human studies [2,39].

**Figure 1:**
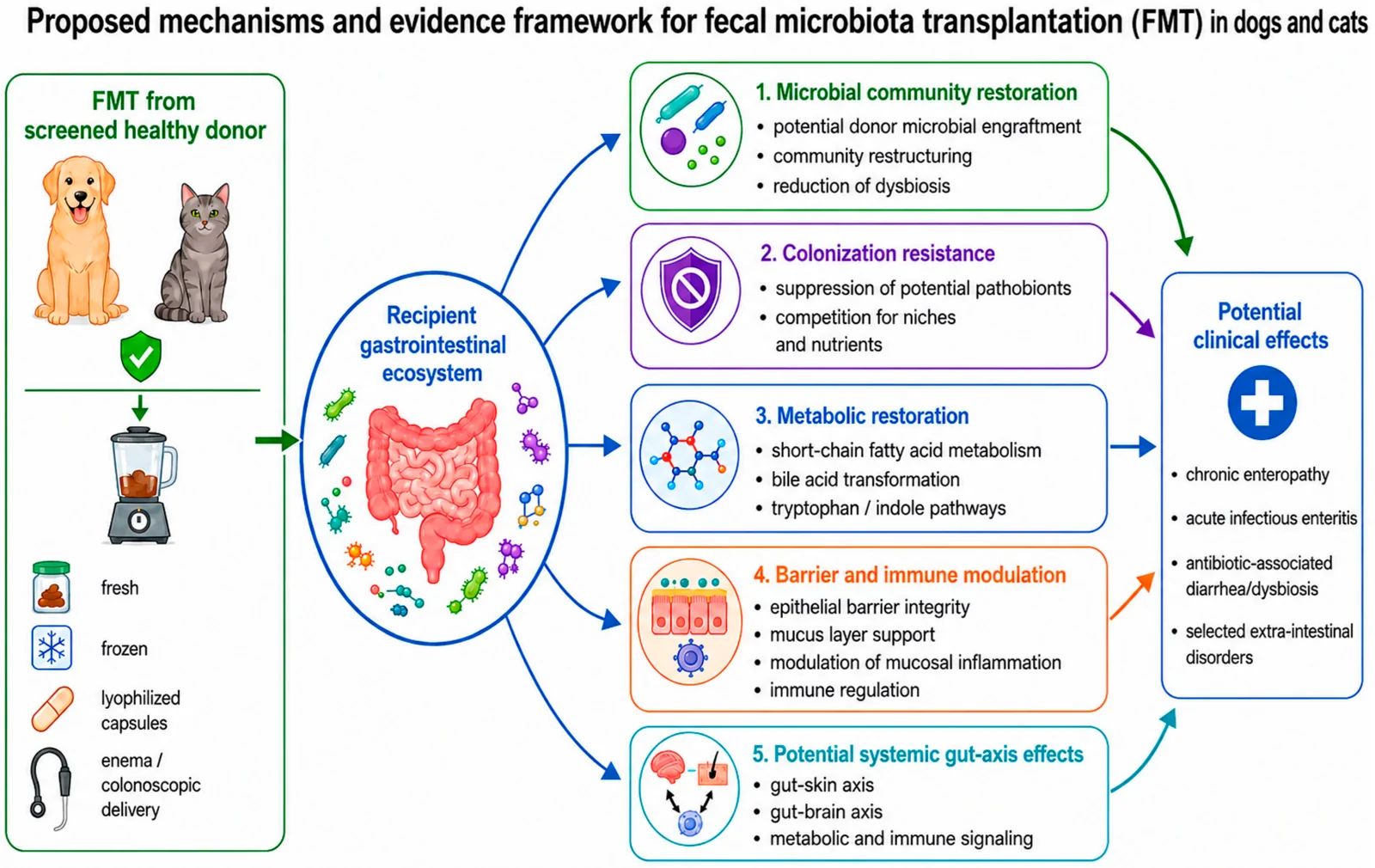
Proposed mechanisms and evidence framework for FMT in dogs and cats.

Interest in FMT for companion animals has increased because the intervention targets the intestinal microbial community as an ecological system rather than a single pathogen or isolated probiotic strain. Potential therapeutic effects include donor microbial engraftment, suppression of pathobionts, restoration of microbial metabolites, improved epithelial barrier function, immune modulation, and systemic signaling through gut–skin and gut–brain axes [39,40,41,42,43,44,45,46,47,48,49]. However, most veterinary studies remain small, uncontrolled, or heterogeneous in design, and interpretation is further limited by concurrent treatments, lack of blinding, reliance on subjective or owner-reported outcomes in some studies, incomplete follow-up, and substantial variation in donor selection, FMT preparation, route of administration, dose, and treatment frequency. These factors make it difficult to attribute clinical improvement specifically to FMT. FMT may influence recipient health through microbial community restructuring, colonization resistance, metabolic restoration, barrier and immune modulation, and extra-intestinal gut–skin or gut–brain signaling. The strength of evidence for these pathways varies by species, indication, formulation, delivery route, and study design, and they should therefore not be regarded as equally established mechanisms. These proposed mechanisms and their varying levels of evidential support are summarized in Figure 1.

Previous reviews of FMT in companion animals have generally been limited by species, clinical indication, or emphasis on microbiome mechanisms, and few have integrated recent clinical evidence with developments in treatment delivery, preparation, safety, and standardization. Importantly, the veterinary FMT literature has also evolved substantially over time. Earlier evidence was dominated by individual case reports, small case series, and predominantly rectal administration, whereas more recent studies increasingly include prospective cohorts, randomized or controlled trials, repeated-treatment protocols, and orally administered lyophilized preparations. Outcome assessment has likewise expanded from predominantly clinical observations to include validated disease activity indices, dysbiosis measures, microbiome profiling, and donor–recipient microbial engraftment. Recent studies have also broadened the range of investigated indications, while providing more systematic information on tolerability, donor screening, and treatment standardization. The present review therefore not only synthesizes the current evidence for FMT in dogs and cats but also examines how the field has evolved with respect to clinical indications, routes and formulations of administration, treatment protocols, clinical and microbiome-related responses, adverse effects, and methodological rigor. By explicitly distinguishing controlled from uncontrolled evidence and identifying changes in both clinical application and study design, we aim to clarify which developments may already be relevant to clinical decision-making and which should primarily inform future research. The principal contribution of this review is thus a cross-species and temporally contextualized framework linking clinical outcomes, mechanistic plausibility, product standardization, and safety considerations.

## 2. Materials and Methods

### 2.1. Review Design and Scope

This article was designed as a structured narrative review of FMT in companion animals, with primary emphasis on dogs and cats. The review aimed to synthesize clinically relevant evidence while also integrating selected mechanistic, methodological, and safety literature needed to interpret the veterinary FMT field. Because the available studies varied substantially in design, indication, delivery route, dosing protocol, and outcome measures, a meta-analysis was not appropriate. The review was not registered as a systematic review protocol and did not include a formal risk-of-bias assessment.

Eligible publications included peer-reviewed original studies, controlled trials, cohort studies, case series, case reports, microbiome or metabolomic studies, safety reports, clinical guidelines, preparation protocols, and relevant reviews involving FMT in dogs, cats, or companion-animal contexts. Studies focused exclusively on livestock, laboratory animals, or wildlife were excluded unless they provided methodological or safety information directly relevant to companion-animal FMT. Conference abstracts without full peer-reviewed manuscripts and studies limited to probiotics, prebiotics, antibiotics, or diet without FMT were excluded from the core FMT evidence synthesis, although selected non-FMT microbiome interventions were discussed for comparative context.

### 2.2. Literature Search and Evidence Synthesis

Literature was identified through structured searches of PubMed/MEDLINE, PubMed Central, and Web of Science for publications from January 2005 to May 2026, with the final search conducted in May 2026. Search terms combined FMT-related terminology with species and disease terms, including “fecal microbiota transplantation”, “faecal microbiota transplantation”, “fecal microbial transplantation”, “faecal microbial transplantation”, “FMT”, “fecal transplant”, “stool transplant”, “dog”, “canine”, “cat”, “feline”, “companion animal”, “chronic enteropathy”, “chronic inflammatory enteropathy”, “protein-losing enteropathy”, “dysbiosis”, “diarrhea”, and “donor screening”. Database-specific syntax was adjusted according to the indexing and search functions of each platform, while the same core concepts and eligibility framework were retained across databases. Database-specific search strategies were developed for each platform based on the following core concepts: (“fecal microbiota transplantation” OR “faecal microbiota transplantation” OR FMT OR “fecal transplant” OR “stool transplant”) AND (dog OR canine OR cat OR feline OR “companion animal”) AND (enteropathy OR diarrhea OR dysbiosis OR “donor screening” OR safety). Reference lists of key reviews, clinical studies, and companion-animal FMT guidelines were screened manually to identify additional relevant publications. The core clinical evidence synthesis included 22 selected companion-animal FMT clinical reports or studies, including 17 canine and 5 feline studies. Additional clinical reports were discussed where relevant to specific applications, administration routes, or methodological considerations.

Screening, study selection, and data extraction were performed by the authors. The included studies were peer-reviewed original clinical reports describing FMT administration in dogs or cats within the predefined publication period. Conference abstracts without a full peer-reviewed manuscript, studies not involving FMT, and non-clinical reviews or methodological papers were excluded from the core clinical study count. Eligibility was not determined by study outcome, sample size, administration route, or completeness of reporting. For included studies, species, indication, study design, FMT preparation and route, clinical and microbiome outcomes, adverse events, follow-up, and major limitations were extracted where reported. Evidence was classified descriptively, based on study design, presence or absence of a control group, sample size, follow-up duration, objective microbiome or metabolomic endpoints, and susceptibility to confounding by concurrent therapy. The following narrative evidence categories were used throughout the review: controlled veterinary clinical evidence, uncontrolled veterinary clinical evidence, mechanistic veterinary evidence, and extrapolated or hypothesis-generating evidence. No formal risk-of-bias tool or GRADE assessment was applied; therefore, evidence labels in the text and tables should be interpreted as descriptive evidence categories rather than formal certainty ratings.

## 3. The Companion-Animal Gut Microbiome: Health, Dysbiosis, and Diagnostic Assessment

### 3.1. Healthy Canine and Feline Microbiomes

Culture-independent sequencing of fecal samples has shown that healthy dogs exhibit a range of fecal microbial profiles commonly characterized by Firmicutes, Fusobacteria, Bacteroidetes, Proteobacteria, and Actinobacteria [50,51]. *Fusobacterium* is a notable example of species-specific interpretation: in dogs, it is often abundant in healthy fecal communities and may contribute to protein and amino acid fermentation, whereas in humans, some *Fusobacterium* species are more often discussed in disease contexts [2,50]. Large-scale canine sequencing studies have refined species-level definitions of core taxa and highlighted the influence of diet, age, and breed on microbial structure [52,53,54]. Importantly, fecal microbial profiles should not be assumed to fully represent mucosa-associated or more proximal intestinal communities.

Cats share some dominant bacterial phyla with dogs but differ in microbial proportions and metabolic context, consistent with their obligate carnivory [1,55,56]. These differences are clinically important because donor screening, donor–recipient matching, and outcome interpretation should be species-specific. A donor profile considered healthy for a cat should not be assumed to restore a healthy canine microbiome, and vice versa.

### 3.2. Dysbiosis and Functional Consequences

Dogs with chronic inflammatory enteropathy (CIE) commonly show expansion of Proteobacteria, including *Escherichia coli*, depletion of Fusobacteria, and reductions in Firmicutes taxa within Clostridia clusters XIVa and IV [57,58,59]. However, increased relative abundance of *E. coli* should not be interpreted as evidence of pathogenicity, because commonly used 16S rRNA sequencing and broad qPCR approaches generally cannot reliably distinguish commensal from pathogenic strains. These compositional changes are accompanied by functional disturbances, including reduced fecal concentrations of acetate, propionate, and total short-chain fatty acids, as well as altered bile acid conversion due in part to depletion of *Clostridium hiranonis* [8,9]. However, fecal short-chain fatty acid concentrations do not directly reflect intestinal production because they are also influenced by host absorption, microbial utilization, intestinal transit, diet, and stool water content. Alterations in short-chain fatty acid availability may therefore contribute to impaired epithelial and immune homeostasis, although fecal concentrations alone cannot establish reduced intestinal production [8,60,61,62,63,64].

Feline dysbiosis is less extensively characterized, but cats with chronic enteropathy, particularly inflammatory phenotypes, or small-cell intestinal lymphoma show microbial patterns that may have diagnostic and prognostic relevance [20,21,65,66,67]. Antibiotic-associated dysbiosis is also clinically relevant in companion animals. Metronidazole can disrupt microbiome composition and metabolic profiles beyond the treatment period, creating a rationale for interventions that restore microbial function rather than merely resolving diarrhea [68,69].

### 3.3. Dysbiosis Index as a Clinical and Research Tool

The qPCR-based Dysbiosis Index (DI) provides a practical summary measure of changes in a predefined bacterial panel associated with fecal dysbiosis. It does not capture the entire intestinal microbiome or all forms of dysbiosis and should therefore be interpreted as a complementary rather than comprehensive microbiome measure. In dogs, the DI uses selected bacterial targets and total bacterial abundance and has shown diagnostic utility in distinguishing dogs with CIE from healthy dogs [70]. The DI has become a common endpoint in canine FMT studies and may help identify patients more likely to respond; several studies suggest that baseline dysbiosis severity is associated with treatment outcome [71,72]. A feline DI has also been developed and is increasingly used in feline microbiome studies [73].

**Figure 2:**
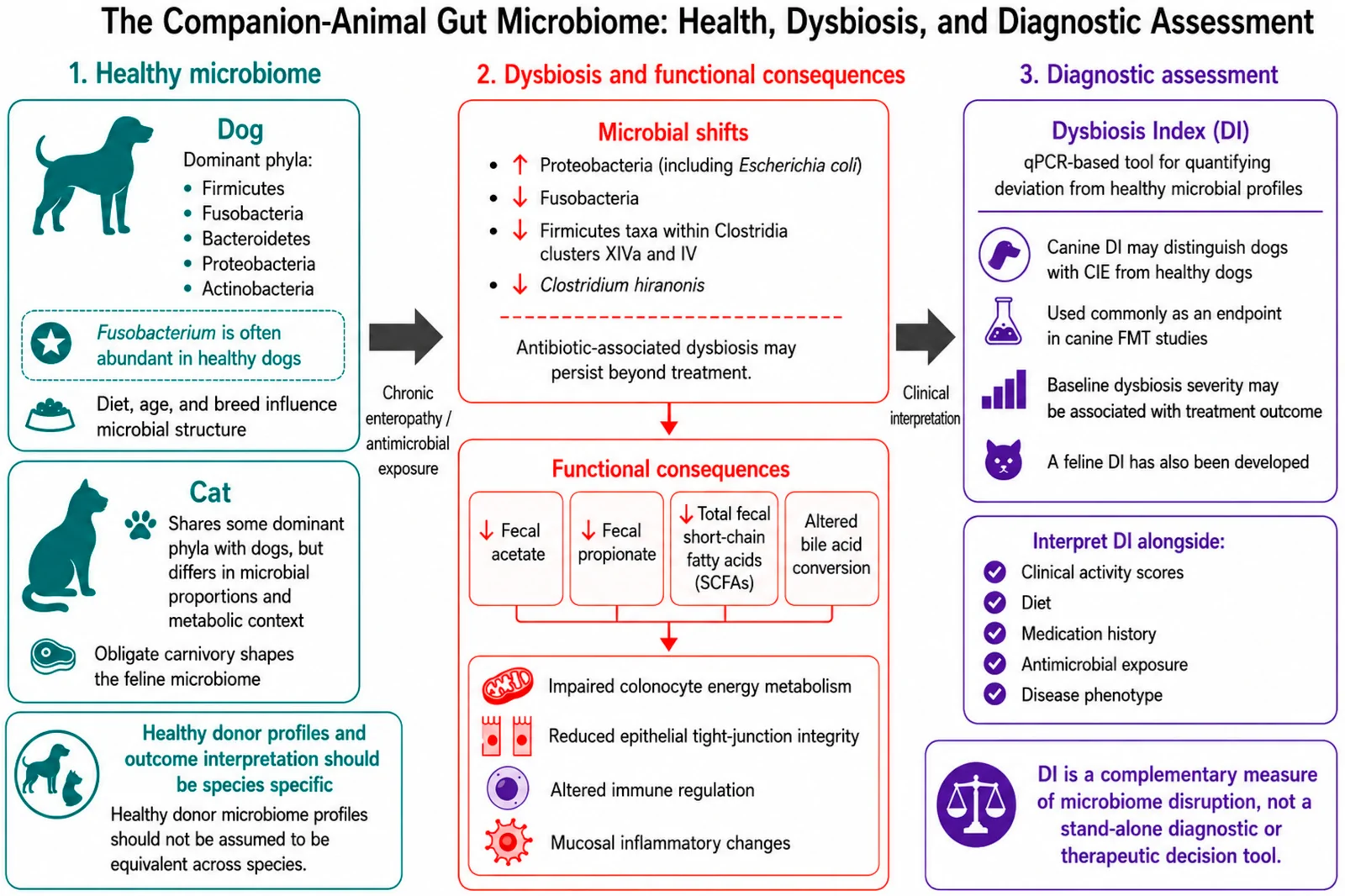
Species-specific healthy gut microbiomes, dysbiosis-associated functional disruption, and diagnostic assessment in dogs and cats.

These tools are valuable because they provide objective endpoints beyond owner-reported clinical signs. However, DI results should be interpreted alongside clinical activity scores, diet, medication history, antimicrobial exposure, and disease phenotype. Canine and feline fecal microbiomes share several dominant bacterial phyla but differ in microbial proportions and metabolic context, supporting species-specific interpretation of donor profiles and FMT outcomes. Dysbiosis may involve shifts in bacterial composition together with altered short-chain fatty acid concentrations, bile acid conversion, epithelial barrier function, and immune regulation. These relationships are summarized in Figure 2.

## 4. FMT in Canine Medicine

Canine FMT evidence has expanded from isolated case reports to prospective cohorts, multicenter studies, and small randomized trials. Table 1 summarizes selected clinical studies and indicates study design as a practical evidence-level marker. Overall, the clinical literature is most developed for canine chronic enteropathy (CE), although the enrolled populations vary in diagnostic characterization, and histopathological confirmation of intestinal inflammation was not required or available in all studies. Most available evidence remains observational, uncontrolled, or underpowered, and controlled findings are still limited and heterogeneous. The strongest controlled evidence comes from selected acute infectious settings, particularly canine parvoviral enteritis, whereas evidence for other acute diarrheal syndromes remains mixed.

### 4.1. Chronic Enteropathy and Related Phenotypes

The most extensively studied canine indication for FMT is chronic enteropathy (CE), although the diagnostic work-up and availability of histopathological confirmation vary among studies. Retrospective and prospective studies have reported reductions in CIBDAI or related disease activity scores after FMT, particularly in protocols involving repeated administrations [71,72,86]. Toresson et al. reported clinical improvement in 31 of 41 dogs with CE receiving adjunctive enema-based FMT and identified lower baseline Dysbiosis Index (DI) as a potential predictor of response [71]. Vecchiato et al. subsequently reported clinical improvement in 17 of 20 dogs with diet-refractory CE in a prospective multicenter study, with median CIBDAI decreasing from 5 to 1 [86]. In a prospective longitudinal study of dogs with refractory CE, repeated FMT was associated with clinical response in 28 of 39 dogs and reduced corticosteroid use in a subset of cases [72]. Microbiome-related endpoints, including changes in the DI, bacterial abundance, or donor–recipient ASV sharing, should be interpreted separately from clinical outcomes and do not by themselves demonstrate clinical efficacy.

Oral lyophilized capsules may improve practicality by enabling repeated outpatient dosing. Brugnoli et al. reported an 82% positive clinical response among 111 analyzable dogs with CE treated with oral lyophilized FMT capsules from an initial prospective multicenter cohort of 171 dogs [82]. This result is clinically encouraging, but the uncontrolled design, substantial missing post-treatment data, and lack of a placebo control mean that causal interpretation should remain cautious.

Randomized evidence remains limited. Collier et al. randomized 13 dogs with histopathologically confirmed CIE, reported as IBD in the original publication, to FMT or placebo in addition to standard therapy. CCECAI decreased over time in both groups, with a numerically greater reduction in the FMT group, but no significant between-group difference was detected [79]. Hanifeh et al. provided double-blind proof-of-concept evidence in dogs with tylosin-responsive enteropathy (using the treatment-response terminology of the original study), reporting fewer relapses after oral FMT capsules than after placebo, although the sample size was small [83].

Taken together, current canine data support further investigation of FMT as a microbiome-directed adjunct, but do not yet establish definitive clinical efficacy. Most positive findings derive from observational or small studies, and causal interpretation remains limited by heterogeneous protocols, concurrent treatments, and limited controlled replication. Larger sham-controlled trials with standardized donor screening, dose-finding, controlled background therapy, objective microbiome endpoints, and relapse-free survival outcomes are needed before FMT can be recommended as routine treatment for canine CE.

### 4.2. Acute Diarrhea, Parvovirus, and Acute Hemorrhagic Diarrhea Syndrome

The strongest controlled canine evidence in acute gastrointestinal disease comes from canine parvoviral enteritis. In a randomized controlled trial of 66 puppies, enema-based FMT accelerated diarrhea resolution and shortened hospitalization compared with standard care alone [74]. This finding is clinically important because it used meaningful clinical endpoints in a high-morbidity infectious disease context.

In non-specific acute diarrhea, a prospective non-randomized treatment trial comparing FMT with metronidazole found that clinical signs improved in both groups, while microbiome recovery was more evident after FMT [76]. This distinction highlights the difference between symptomatic resolution and microbiome recovery. Conversely, a pilot study in acute hemorrhagic diarrhea syndrome found no additional benefit from a single enema-based FMT over standard supportive care [78]. These mixed findings suggest that timing, disease mechanism, dose, frequency, and route of administration may strongly influence outcomes in acute gastrointestinal disease.

### 4.3. Extra-Intestinal Applications

Early studies have explored FMT beyond gastrointestinal disease. Sugita et al. reported improved CADESI-04 scores after a single oral FMT in dogs with atopic dermatitis [81], and Watanangura et al. reported changes in behavioral comorbidities and urinary neurotransmitter profiles in dogs with drug-resistant epilepsy [89]. These findings are hypothesis-generating rather than practice-changing, but they are consistent with broader concepts of gut–skin and gut–brain axis signaling [2,45].

Extra-intestinal applications should currently be considered exploratory. Future studies should include randomized controls, validated dermatological or neurological endpoints, control of concurrent medications, metabolomic assessment, and longitudinal microbiome profiling to determine whether clinical changes are reproducible and mechanistically linked to FMT.

### 4.4. Oral Lyophilized FMT as a Practical Advance

Fresh or frozen enema-based FMT can deliver a high microbial dose directly to the distal gut, but it often requires restraint, sedation, immediate product handling, or referral-center infrastructure. Oral lyophilized capsules address several practical limitations by allowing repeated outpatient or owner-administered dosing under veterinary supervision, easier storage, and batch-level quality control. Pilot work supports feasibility and tolerability in dogs [90], and capsule-based studies have demonstrated measurable donor microbial signals [83,84]. Key unresolved questions include optimal dose, treatment duration, capsule viability, donor selection, engraftment consistency, and whether passage through the upper gastrointestinal tract alters efficacy compared with rectal administration.

## 5. FMT in Feline Medicine

Feline FMT research remains very limited, with few clinical studies and limited controlled evidence. Available studies are constrained by small sample sizes, heterogeneous indications, uncontrolled designs, subjective or owner-reported outcomes in some studies, and incomplete follow-up. Although microbiome changes have been reported after FMT, these findings should not be interpreted as evidence of therapeutic benefit, and clinical efficacy has not yet been established. Table 2 summarizes selected feline reports and trials and distinguishes controlled from uncontrolled evidence.

### 5.1. Chronic Enteropathy and Chronic Digestive Signs

The first documented feline case report described sustained remission in a cat with refractory ulcerative colitis after two enema-based FMT procedures [91]. Larger experience has come from oral capsule studies in cats with chronic vomiting, diarrhea, or constipation. Rojas et al. reported microbiome-level changes in 46 cats with chronic digestive signs, including donor–recipient shared ASVs and shifts toward healthier reference profiles in some animals [92]. Because the study was uncontrolled and primarily focused on microbiome outcomes, these findings should not be interpreted as evidence of clinical efficacy.

The first prospective, blinded, controlled feline chronic enteropathy trial did not demonstrate significant improvement in Dysbiosis Index (DI) or Feline Chronic Enteropathy Activity Index (FCEAI) after a single enema-based FMT compared with control treatment [93]. This negative controlled result is important because it prevents overinterpretation of uncontrolled feline case reports and observational cohorts. The FCEAI provides a relevant clinical activity measure [96], whereas the diagnostic overlap among feline inflammatory and low-grade neoplastic enteropathies remains an important interpretive challenge [97]. Possible explanations for the neutral FMT result include insufficient sample size, single-dose exposure, enema-only delivery, concurrent standard therapy in both groups, and heterogeneity among feline CE phenotypes. Future feline trials should therefore evaluate repeated dosing, oral versus rectal delivery, phenotype stratification, and longer follow-up before efficacy claims are made.

Accordingly, current feline evidence should be interpreted as supporting biological activity and short-term tolerability rather than established clinical efficacy. Microbiome changes alone should not be considered evidence of therapeutic benefit, and the available evidence is insufficient to establish long-term safety. Routine clinical use in cats should remain cautious and preferably evidence-generating until repeated-dose, sham-controlled, phenotype-stratified trials with longer follow-up are available.

### 5.2. Experimental Recovery from Antibiotic-Associated Dysbiosis

A randomized experimental study in 25 healthy adult cats treated with metronidazole found that FMT was the only intervention to normalize the feline Dysbiosis Index (DI) immediately after the intervention, although this effect appeared transient in some cats [95]. Because this study used experimentally induced antibiotic-associated dysbiosis in healthy cats rather than naturally occurring chronic enteropathy, its findings support a microbiome-level biological effect of FMT but should not be interpreted as evidence of clinical efficacy in cats with chronic enteropathy. Future clinical studies should evaluate repeated dosing, oral versus rectal delivery, disease phenotype stratification, and long-term follow-up after antimicrobial discontinuation.

### 5.3. Safety and Donor Screening in Cats

Available feline safety data are limited. Lee et al. reported predominantly grade I–II adverse events after enema-based FMT in nine cats, with one grade III event involving severe abdominal pain; most adverse events resolved with supportive care or no intervention [94]. The controlled feline CE trial also reported tolerability without serious adverse events [93]. Feline donor screening should be stricter than canine screening in several respects, including testing for feline immunodeficiency virus, feline leukemia virus, and *Tritrichomonas foetus*, in addition to bacterial pathogens, parasites, and antimicrobial-resistant organisms [98].

## 6. Preparation, Standardization, and Clinical Guidelines

### 6.1. Donor Screening and Safety Safeguards

Donor screening is a key component of FMT safety, but safe clinical use also depends on appropriate recipient selection, standardized processing, batch quality control and traceability, appropriate administration, and systematic adverse-event monitoring. Companion-animal FMT guidelines recommend screening for enteric bacterial pathogens such as *Salmonella*, *Campylobacter*, *Clostridioides difficile*, *Clostridium perfringens*, and extended-spectrum beta-lactamase-producing *Escherichia coli*; parasites such as *Giardia* and *Cryptosporidium*; and species-specific viral or protozoal pathogens where relevant [98,99,100]. Culture-based screening, targeted molecular assays, and broader resistome analysis provide complementary but non-equivalent information; therefore, a negative selective culture does not exclude all resistant organisms or antimicrobial-resistance genes. Donor programs should define a minimum screening panel, periodic re-screening intervals, exclusion criteria, and procedures for positive results, including temporary or permanent donor exclusion and re-evaluation before further use. Donors should also be clinically healthy, free of recent antimicrobial exposure, and maintained on a stable diet.

At a minimum, future clinical protocols should report donor health criteria, recent antimicrobial exposure, diet stability, screening panels, re-screening intervals, fecal handling time, storage conditions, batch identifiers, recipient monitoring, and adverse-event grading. For cats, donor programs should explicitly include feline-specific infectious disease screening in addition to the enteric pathogen and antimicrobial-resistance safeguards used across companion animals.

Because FMT transfers a complex and incompletely characterized biological product, negative pathogen tests cannot eliminate all risk. Safety programs should therefore include donor re-screening intervals, batch traceability, recipient monitoring, adverse-event reporting, and, where feasible, antimicrobial-resistance gene surveillance.

### 6.2. Fresh, Frozen, and Lyophilized Preparations

Microbial DNA abundance does not necessarily reflect viable microbial load, and preservation of overall viability may still be accompanied by selective loss of sensitive taxa. Therefore, microbial viability and product standardization depend not only on whether a preparation is fresh, frozen, or lyophilized, but also on processing conditions, storage, oxygen exposure, and product-specific validation. Fresh preparations may maximize viability but require rapid processing and administration. Frozen preparations improve scheduling flexibility but require validated cryoprotectants and cold-chain storage. Lyophilized preparations offer the greatest practical standardization and support oral capsule delivery, although viability, dose equivalence, engraftment consistency, and long-term stability require continued validation [101]. The practical differences among fresh, frozen, and lyophilized FMT preparations are summarized in Table 3.

### 6.3. Rectal/Enema Administration of FMT

Rectal or enema administration is one of the commonly used routes of FMT in veterinary medicine and has been applied in both dogs and cats. In addition to the studies discussed above, Ural et al. reported extensive clinical experience with predominantly rectal FMT in dogs with various disease conditions [102], while rectal FMT has also been investigated in cats with ataxia [103]. These studies provide additional clinical experience regarding the feasibility of rectal FMT, although further controlled studies are required to establish its efficacy across different indications.

### 6.4. Regulatory and Commercial Standardization

The field is moving from case-by-case clinical preparation toward standardized protocols and commercial capsule products. This transition may improve reproducibility, quality control, accessibility, and treatment adherence, but it also raises important regulatory questions. Veterinary FMT products do not yet have a clearly harmonized regulatory pathway, and their classification as biological products, veterinary medicinal products, supplements, or compounded preparations remains context dependent. Clear product classification, minimum donor screening requirements, batch-release criteria, labeling standards, adverse-event reporting systems, and antimicrobial-resistance surveillance are needed before broader clinical adoption [98,101,104].

## 7. Mechanisms of Action

**Figure 3:**
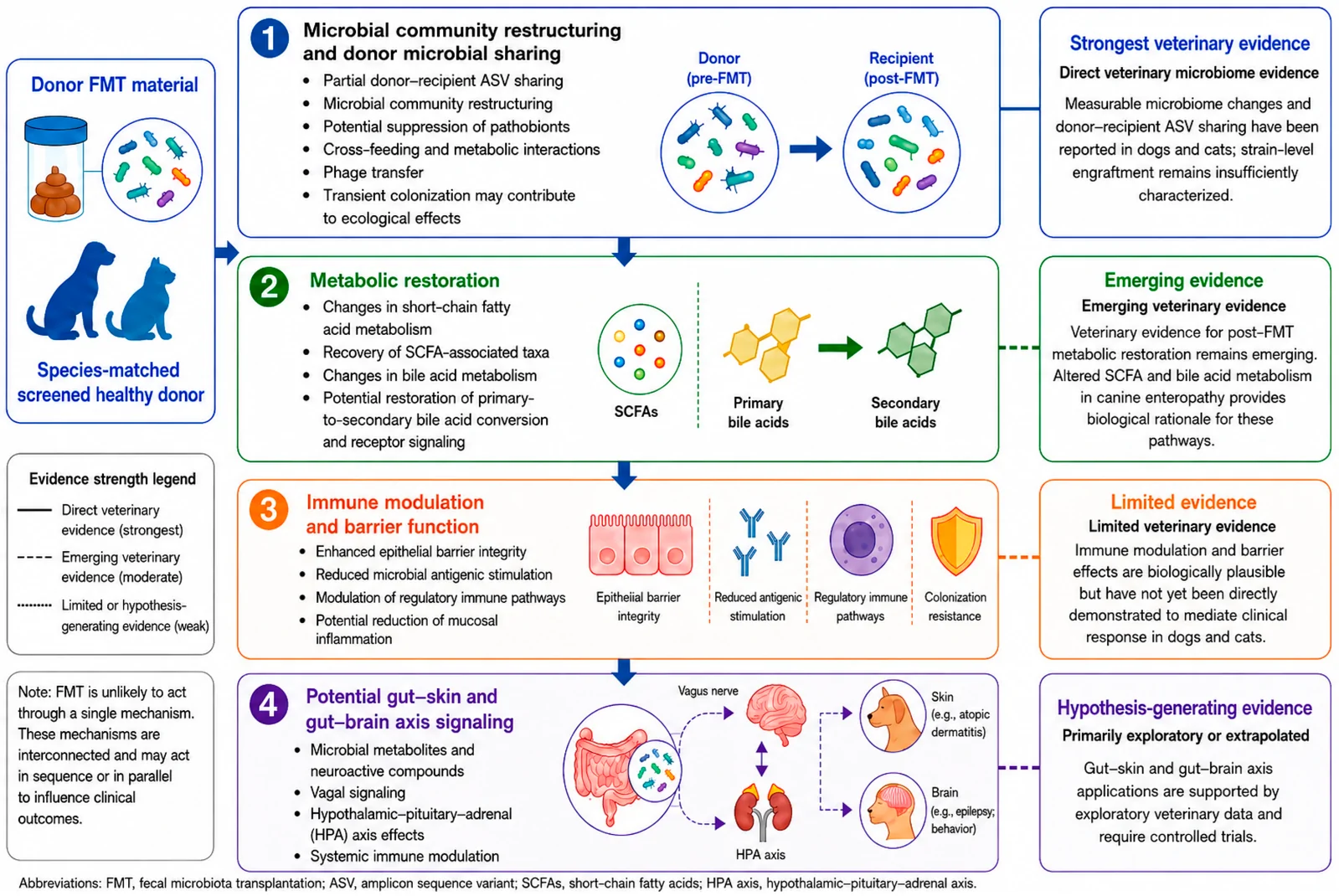
Proposed multi-level mechanisms of FMT in companion animals.

FMT is unlikely to act through a single mechanism. A multi-level model is more plausible: donor microorganisms may partially engraft, recipient microbial communities may be restructured, microbial metabolic functions may recover, barrier and immune functions may improve, and systemic signaling may influence extra-intestinal outcomes. However, the evidentiary basis differs across these mechanisms. In companion animals, direct support is strongest for measurable microbiome shifts and is emerging for metabolic endpoints, whereas immune modulation and gut–skin or gut–brain signaling remain supported mainly by human, experimental, or hypothesis-generating veterinary data. In companion animals, direct evidence is strongest for measurable microbiome shifts and donor–recipient microbial sharing, emerging for metabolic restoration, and limited or hypothesis-generating for immune-mediated and extra-intestinal effects. These interconnected mechanisms and their differing levels of evidence are summarized in Figure 3. Post-treatment microbiome changes should not be assumed to be donor-derived or mechanistically responsible for clinical improvement, particularly in uncontrolled studies in which diet, antimicrobial withdrawal, concurrent treatment, spontaneous recovery, and temporal variation may also contribute.

### 7.1. Microbial Engraftment and Community Restructuring

Strain-level, longitudinal evidence provides the strongest support for donor engraftment, whereas shared ASVs alone should not be considered confirmation of engraftment. Human metagenomic studies show that donor strain engraftment varies by disease, donor–recipient compatibility, and microbial taxa, and that higher engraftment can be associated with better outcomes [40]. Veterinary data are emerging: oral FMT studies have reported measurable donor ASV sharing in dogs and cats, although rates were modest [84,92]. Shared ASVs may represent multiple strains, pre-existing taxa below the detection threshold, or taxa common in the wider population. Partial engraftment should not be dismissed, because ecological effects may also arise through transient colonization, cross-feeding, phage transfer, metabolite shifts, and suppression of pathobionts [41]. Accordingly, post-treatment community shifts, demonstrable donor-derived engraftment, and microbiome changes that mediate clinical improvement should be regarded as distinct levels of evidence.

### 7.2. Metabolic Restoration

Metabolic restoration may be as important as compositional change. Dogs with CIE show reduced fecal short-chain fatty acids and altered bile acid metabolism [8,9]. Recovery of short-chain fatty acid-producing organisms may support colonocyte energy supply, epithelial tight-junction integrity, and regulatory immune pathways. Recovery of *Clostridium hiranonis* or other bile acid-converting organisms may influence primary-to-secondary bile acid conversion and inflammation-related receptor signaling [2,9,48,105,106,107]. However, species-specific bile acid pools and diet-associated fermentation patterns mean that canine, feline, and human mechanisms cannot be assumed to be identical.

### 7.3. Immune Modulation and Barrier Function

FMT may reduce mucosal immune activation by increasing colonization resistance, decreasing pathogen-associated antigenic stimulation, improving epithelial barrier function, and promoting regulatory immune pathways [22,42,43,44,61,108,109,110,111,112]. In dogs and cats, however, direct evidence that these immunological changes mediate clinical response remains limited. Future veterinary studies should include mucosal cytokine profiling, regulatory T-cell markers, secretory immunoglobulin A, histopathology, metabolomics, and longitudinal clinical endpoints to test whether immunological changes precede or simply accompany clinical improvement.

### 7.4. Gut–Skin and Gut–Brain Axis Signaling

Exploratory canine studies in atopic dermatitis and epilepsy are consistent with gut–skin and gut–brain axis hypotheses [81,89]. Candidate mediators include microbial metabolites, neuroactive compounds, vagal signaling, hypothalamic–pituitary–adrenal axis effects, and systemic immune modulation [2,45,49]. These applications should be treated as mechanistic leads rather than established indications until controlled trials replicate clinical benefits and define biomarkers of response.

## 8. FMT Compared with Other Microbiome-Modulating Therapies

### 8.1. Probiotics and Prebiotics

Probiotics and prebiotics are easier to regulate, administer, and standardize than FMT, but their effects are typically strain-specific, substrate-specific, condition-specific, and often transient [18,113,114,115,116,117]. Some probiotic trials in dogs and cats have reported benefits for acute diarrhea, fecal consistency, or selected gastrointestinal outcomes, but systematic reviews generally rate the evidence as limited, heterogeneous, or of variable clinical relevance [113,114,118,119]. Prebiotics may support endogenous microbial activity through selective substrate provision, but they do not directly replace a disrupted microbial community. FMT differs conceptually because it transfers a complex microbial ecosystem, including multiple viable taxa and associated functional capacity, rather than one or a few selected organisms or substrates. However, direct comparisons should be made cautiously because the probiotic evidence base includes systematic reviews and controlled trials, whereas much of the veterinary FMT evidence remains based on small or uncontrolled studies. Both approaches should therefore be evaluated using comparable criteria, including study quality, effect size, safety, durability, and clinical relevance.

### 8.2. Postbiotics, Phage Therapy, and Defined Consortia

Postbiotics, bacteriophage therapy, and defined microbial consortia may eventually offer more precise and standardized alternatives to whole-stool FMT [48,110,120,121,122,123,124]. Postbiotics avoid the risks associated with transfer of live organisms, phages can be designed or selected to target specific bacterial pathogens, and defined consortia can be manufactured with known composition and quality-control specifications. Whether the ecological complexity of whole-community FMT provides a clinically meaningful advantage over more targeted microbiome-based approaches remains uncertain. Comparative studies are needed to determine which patients require broad ecosystem-level intervention and which may respond adequately to targeted microbiome-derived products.

### 8.3. Practical Clinical Positioning

At present, documented dysbiosis, refractory or relapsing gastrointestinal disease, antibiotic-associated dysbiosis, and incomplete response to appropriate diet or conventional therapy should be regarded as potential clinical contexts for investigation rather than validated indications for FMT. Similarly, the severity or normalization of the Dysbiosis Index should not be assumed to predict or confirm clinical response. FMT should not replace diagnostic work-up, dietary trials, parasite control, antimicrobial stewardship, or immunosuppressive treatment when these are indicated. When used outside controlled trials, clinicians should apply standardized donor screening, batch traceability, adverse-event monitoring, and owner-informed consent, and should frame the intervention as evidence-generating rather than definitively established for most indications. In clinical research, microbiome endpoints such as the Dysbiosis Index should be interpreted together with disease activity scores, medication reduction, relapse-free survival, adverse-event monitoring, and quality-of-life measures.

## 9. Knowledge Gaps and Future Directions

### 9.1. Main Evidence Gaps

The central evidence gap is the lack of adequately powered, randomized, sham-controlled trials evaluating FMT in canine and feline CE. Existing studies are often small, uncontrolled, retrospective, or confounded by concurrent standard therapy [71,72,79,82,88,93]. In canine studies, additional heterogeneity arises from differences in diagnostic work-up and the inconsistent availability of histopathological confirmation of intestinal inflammation. This limitation is particularly important in feline medicine, where the number of controlled studies and enrolled animals remains small. Dosing and delivery protocols are also largely empirical. Published studies differ in the use of fresh, frozen, or lyophilized material; enema versus oral administration; single versus repeated dosing; donor selection; background diet; concurrent medications; and duration of follow-up.

Long-term safety remains insufficiently characterized. Most veterinary studies report outcomes over days to months, and only isolated reports provide longer follow-up [80]. Key unresolved issues include the durability of donor microbial engraftment, timing and predictors of relapse, effects on antimicrobial-resistance gene carriage, risk of zoonotic pathogen transfer, and safety in immunocompromised patients or animals with severe intestinal inflammation. The welfare and ethical management of donor animals also warrant consideration, particularly where donor eligibility may involve restrictive housing, dietary control, or repeated fecal collection.

### 9.2. Research Priorities

Priority 1: Adequately powered, sham-controlled randomized trials in canine and feline CE, with standardized background therapy, clearly defined diagnostic criteria, phenotypic stratification, explicit reporting of histopathological confirmation where available, and prespecified clinically meaningful endpoints.

Priority 2: Head-to-head route and dosing studies comparing enema, oral capsule, and combined protocols while controlling microbial dose, viability, dosing frequency, and treatment duration.

Priority 3: Multi-omics mechanistic studies integrating metagenomics, metabolomics, bile acid profiling, short-chain fatty acid measurement, immunophenotyping, and validated clinical activity scores.

Priority 4: Donor optimization studies comparing universal donors, pooled donors, diet-matched donors, microbiome-defined donors, and pathogen- and antimicrobial-resistance-screened donor programs, while also considering donor welfare and ethical management.

Priority 5: Long-term safety surveillance extending to at least 12 months, including relapse, adverse events, antimicrobial-resistance markers, microbiome durability, and owner-reported quality-of-life outcomes.

### 9.3. Reporting Standards and One Health Considerations

Veterinary FMT studies should report donor criteria, screening tests, stool handling time, processing method, storage conditions, microbial dose or a justified proxy, viability data where available, route of administration, dosing frequency, recipient diet, concurrent medications, adverse-event grading, and microbiome endpoints. The GRAFT framework for animal fecal transplantation reporting provides useful principles, although companion-animal-specific extensions may be needed [125].

Because dogs and cats live in close contact with humans, FMT also has a One Health dimension. Donor material should be screened not only to protect the recipient, but also to reduce risks of zoonotic pathogen transmission and antimicrobial-resistance dissemination within households and veterinary environments [98,104]. Responsible clinical translation will therefore require not only efficacy trials, but also standardized reporting, traceable donor screening, long-term safety monitoring, and species-specific guidance for routine veterinary use.

## 10. Limitations of This Review

This review used a structured narrative approach rather than a registered systematic review. Accordingly, no formal risk-of-bias assessment, PRISMA flow diagram, exhaustive search-yield count, or meta-analysis was performed. The synthesis may therefore be influenced by publication bias, selective reporting, and the narrative interpretation of heterogeneous study designs, populations, protocols, and outcome measures. In addition, some recent studies were preliminary in design or had limited follow-up, which restricts conclusions about durability of response and long-term safety. The literature search was limited to PubMed/MEDLINE, PubMed Central, and Web of Science, and independent duplicate screening and data extraction were not performed, which may have increased the potential for subjective study selection or omission of relevant evidence. In addition, limited non-peer-reviewed data were used where relevant, and the substantial heterogeneity in study populations, FMT protocols, outcome measures, and follow-up further limits comparability across studies. Diagnostic characterization also differed among canine CE studies, and histopathological confirmation of intestinal inflammation was not consistently performed or required, limiting direct comparison of broader CE populations with histopathologically confirmed CIE cohorts. Collectively, these limitations reduce confidence in conclusions regarding clinical efficacy, long-term safety, proposed mechanisms, and the appropriate clinical use of FMT.

## 11. Conclusions

FMT is a biologically plausible and increasingly standardized microbiome-directed intervention for companion animals. In dogs, the strongest clinical evidence supports cautious investigation and selected case-by-case adjunctive use in CE, as well as in specific acute infectious settings, particularly canine parvoviral enteritis. However, most canine CE evidence remains observational, underpowered, or confounded by concurrent therapy, and the available studies include heterogeneous disease phenotypes and diagnostic criteria, with histopathological confirmation of intestinal inflammation not consistently available. Definitive efficacy beyond spontaneous remission, contextual effects, and standard treatment has therefore not yet been established. In cats, FMT appears generally well tolerated in the short term, but evidence for clinical efficacy remains unproven. The lack of significant improvement in primary endpoints in the first controlled feline chronic enteropathy trial should temper clinical claims while motivating better-designed studies using repeated dosing, route comparisons, phenotype stratification, and longer safety follow-up.

Mechanistic understanding supports a multi-level model involving microbial engraftment, metabolic restoration, immune modulation, and gut–brain or gut–skin axis signaling, but veterinary-specific validation remains limited. The next phase of the field should prioritize adequately powered sham-controlled trials, standardized product preparation, rigorous donor screening, dose-finding studies, long-term safety surveillance, and harmonized reporting standards. With these safeguards, FMT may become an important adjunct in the veterinary management of microbiome-associated disease; current evidence supports careful, evidence-generating implementation rather than unrestricted routine use or routine first-line use.

## Figures and Tables

**Table 1:** Summary of selected canine FMT clinical studies, with study design reported descriptively.

Study	Design	*n*	Indication	Route	Key Outcome
Pereira et al. [74]	RCT	66	Canine parvovirus enteritis	Enema	Faster diarrhea resolution; shorter hospitalization
Niina et al. [75]	Case report	1	Refractory CE (histopathologically confirmed CIE; reported as IBD in the original study)	Repeated enema	CIBDAI improved from 9 to 4; Proteobacteria decreased
Chaitman et al. [76]	Prospective treatment trial	18	Acute diarrhea	Enema	DI decreased after FMT, whereas dysbiosis persisted after metronidazole at day 28
Niina et al. [77]	Uncontrolled clinical study	9 (16S sequencing in 3)	CE (histopathologically confirmed CIE; reported as IBD in the original study)	Single rectal enema	CIBDAI decreased and *Fusobacterium* increased after FMT
Gal et al. [78]	Pilot study	8	AHDS	Enema	No additional benefit over standard care
Collier et al. [79]	Double-blind randomized clinical trial	13 (7 FMT; 6 placebo)	CE (histopathologically confirmed CIE; reported as IBD in the original study)	Single retention enema	CCECAI decreased over time in both groups; no significant between-group difference
Cerquetella et al. [80]	Case report	1	Relapsing diarrhea	Oral capsules	Clinical improvement with no serious relapses during 18-month follow-up; maintenance prednisolone was continued
Toresson et al. [71]	Retrospective case series	41	CE	Enema	31/41 dogs improved; lower baseline DI was associated with better response
Sugita et al. [81]	Uncontrolled trial	12	Atopic dermatitis	Single oral administration	CADESI-04 improved; exploratory extra-intestinal signal
Brugnoli et al. [82]	Prospective multicenter cohort	171 enrolled; 111 analyzed	CE	Oral lyophilized capsules	82% response among analyzed dogs; missing outcome data and absence of a control group limit inference
Hanifeh et al. [83]	Double-blind RCT	13/14 analyzed	Tylosin-responsive enteropathy	Oral capsules	71.4% non-relapse after FMT vs. 50% after placebo; difference not statistically significant
Rojas et al. [84]	Cohort	54	Chronic gastrointestinal signs	Oral capsules	18% donor–recipient ASV sharing; *Butyricicoccus* increased
Pérez-Accino et al. [85]	Prospective uncontrolled study	7	CE (histopathologically confirmed CIE)	Rectal FMT	Clinical severity decreased after FMT; improvement was not accompanied by consistent longitudinal changes in fecal microbial community composition or diversity
Vecchiato et al. [86]	Prospective multicenter	20	Diet-refractory CE	Enema	Median CIBDAI improved from 5 to 1; 17/20 improved at 3 months
Toresson et al. [72]	Prospective longitudinal	39	Refractory CE	Repeated enemas	28/39 responded; corticosteroid dose reduced in 13 dogs
Schreiber et al. [87]	Case report	1	Protein-losing enteropathy with concurrent protein-losing nephropathy	Repeated enema and oral lyophilized FMT	Repeated FMT was associated with improvement in clinical status, body weight, serum albumin, fecal consistency, and DI; repeated treatment was required to maintain the response
Allerton et al. [88]	Blinded randomized controlled trial	42 (25 FMT; 17 control)	CE	Single retention enema	No significant additional clinical benefit of FMT plus dietary management over dietary management alone for owner-reported improvement, CIBDAI, or fecal score

Abbreviations: AHDS, acute hemorrhagic diarrhea syndrome; ASV, amplicon sequence variant; CADESI-04, Canine Atopic Dermatitis Extent and Severity Index; CE, chronic enteropathy; CIBDAI, Canine Inflammatory Bowel Disease Activity Index; CIE, chronic inflammatory enteropathy; DI, Dysbiosis Index; FMT, fecal microbiota transplantation; IBD, inflammatory bowel disease; RCT, randomized controlled trial. CE is used as the broader clinical term for canine chronic enteropathy populations. CIE is specified where histopathological confirmation of intestinal inflammation was available. The term IBD is retained only where it reflects terminology used in the original publication. Study design is reported descriptively; these labels are not formal GRADE ratings.

**Table 2:** Summary of selected feline FMT studies, with study design reported descriptively.

Study	Design	*n*	Indication	Route	Key Outcome
Furmanski and Mor [91]	Case report	1	Refractory ulcerative colitis	Enema	Sustained remission reported for 11 months
Rojas et al. [92]	Observational microbiome cohort	46	Chronic digestive signs	Oral capsules	~13% donor–recipient ASV sharing; microbiome shifted toward healthy reference profiles in some cats
Karra et al. [93]	Prospective blinded controlled trial	28	Chronic enteropathy	Single enema	Well tolerated; no significant DI or FCEAI improvement versus controls
Lee et al. [94]	Retrospective case series	9	CE or therapy-resistant diarrhea	Repeated rectal enemas	Predominantly grade I–II AEs; one grade III severe abdominal pain event; 8/9 cats showed complete or partial clinical response
Martini et al. [95]	Randomized experimental study	25	Metronidazole-induced dysbiosis in healthy adult cats	Oral FMT capsules plus diet	FMT was the only intervention to normalize DI, although the effect was transient in some cats

Abbreviations: ASV, amplicon sequence variant; CE, chronic enteropathy; DI, dysbiosis index; FCEAI, Feline Chronic Enteropathy Activity Index; FMT, fecal microbiota transplantation; Study design is reported descriptively; these labels are not formal GRADE ratings.

**Table 3:** Practical comparison of FMT preparation methods for companion animals and their likely clinical-use scenarios.

Parameter	Fresh	Frozen	Lyophilized
Viability	Generally highest immediately after processing	High if cryoprotectant and storage are optimized	Good when validated; product-specific
Storage	Hours; immediate use preferred	Usually ultra-low temperature storage	Room temperature or refrigerated storage may be possible depending on product
Convenience	Low; same-day preparation	Moderate; cold chain required	High; capsules and batch production possible
Standardization	Difficult	Moderate	Highest potential
Main limitation	Logistics and donor availability	Storage and thawing effects	Dose equivalence, viability, and regulatory classification

## Data Availability

No new data were created or analyzed in this study. Data sharing is not applicable to this article.
